# HIV Sexual and Drug-Use Risk in Drug-Dependent Pregnant Patients in Comprehensive Drug Treatment

**DOI:** 10.1155/2011/872638

**Published:** 2011-03-22

**Authors:** Hendrée E. Jones, Wendee M. Wechsberg, Kevin E. O'Grady, Michelle Tuten

**Affiliations:** ^1^RTI International, 3040 Cornwallis Road, Research Triangle Park, NC 27709, USA; ^2^Department of Psychiatry, Johns Hopkins University School of Medicine, Baltimore, MD 21224, USA; ^3^Department of Obstetrics and Gynecology, Johns Hopkins University School of Medicine, Baltimore, MD 21224, USA; ^4^Department of Psychology, University of Maryland, College Park, MD 20742, USA

## Abstract

This secondary analysis study investigated HIV sexual and drug-use risk in drug-dependent pregnant patients over the first month postrandomization to reinforcement-based treatment (RBT) (*n* = 47) or usual care (UC) (*n* = 42). Analysis of primary outcomes had indicated that RBT participants spent significantly longer time in treatment and recovery housing than UC participants. The present study examined the ability of 9 risk markers—age, race, estimated gestational age at treatment entry, lifetime substance abuse treatment episodes, history of prostitution charges, history of serious depression, current heroin injection status, current housing status, and current partner substance use—to predict changes in HIV risks. Sexual risk declined for participant subgroups with prostitution-charge histories and unstable housing. Drug-use risk declined for heroin injectors and nondepressed participants. A relationship was found between number of lifetime drug treatment episodes and sexual and drug-use risk. The role of risk markers in the response of drug-dependent pregnant women to drug treatment require attention.

## 1. Introduction

Substance-abusing women in their reproductive years have relatively high HIV seroprevalence rates [1]. Perinatal transmission of HIV accounts for 90% of pediatric HIV infection [2]. Thus, women with substance use disorders who are also pregnant are an important population to reach for both preventive interventions and treatment [3]. Drug abuse treatment itself has been shown to serve as a protective factor for HIV drug use risks and has been shown to prevent HIV in nonpregnant patients. These results come primarily through reduction of HIV drug-risk behavior [4, 5]. As yet these same results have not been reported in pregnant women. Moreover, to the best of our knowledge, the use of drug abuse treatment to reduce HIV sexual risk behaviors has been inconsistent in nonpregnant patients and has not been shown in pregnant women.

Although comprehensive addiction treatment programs for substance-abusing pregnant women have been shown to result in improved maternal and neonatal outcomes compared to no treatment [6], considerably less attention has been focused on examining the impact that comprehensive drug abuse treatment provided during pregnancy may have on reducing HIV sexual and drug-risk behaviors. In fact, to our knowledge, no empirical study has examined the extent to which comprehensive treatment for drug dependence given to drug-dependent pregnant women can reduce either or both primary sources of HIV risk (that is, sexual and drug-risk behaviors) that threaten not only the pregnant women but also their fetuses. 

Moreover, results of studies in nonpregnant substance-abusing populations suggest that a variety of social and psychological factors are predictive of HIV-risk behaviors, including drug use route [3], homelessness [7, 8], drug-using partner [3], psychiatric status [9], and sex worker status [10]. For substance-using pregnant women entering drug abuse treatment, severity of legal problems has been found to significantly predict HIV sexual risk while severity of medical, drug, and legal problems has each been found to explain a significant portion of the unique variance associated with drug-risk behaviors [11]. 

The purposes of this secondary analysis study were twofold. The first purpose was to examine the short-term outcomes of HIV sexual and drug-use risk in drug-dependent pregnant patients randomized to receive either reinforcement-based treatment (RBT), an intensive manualized therapy [12, 13], or treatment-as-usual (TAU) comprehensive care [14] designed to improve drug treatment and neonatal outcomes. The second purpose was to identify the extent to which selected risk markers might be differentially related to change over time in HIV sexual and drug-risk behavior as a result of the impact of comprehensive treatment for substance use.

## 2. Methods

The parent study, described elsewhere [15], was a randomized controlled trial that examined the relative efficacy of comprehensive Usual Care (UC; *n* = 42) alone or enhanced by Reinforcement-Based Treatment (RBT; *n* = 47) in yielding superior treatment results, maternal delivery, and neonatal outcomes in pregnant women with opioid and/or cocaine substance use disorders. Results from the primary outcomes paper [15] indicated that RBT participants spent a longer time in treatment (*P* < .001) and in recovery housing than did UC participants (*P* = .01). However, no significant differences were found between the RBT and UC conditions in proportion of participants testing positive for any illegal substance. Finally, neonates in the RBT condition spent, on average, fewer hospitalization days after birth than did neonates in the UC condition (*P* = .03), although the two conditions were not significantly different in terms of neonatal gestational age at delivery, birth weight, or number of days hospitalized. 

The parent study was approved by the Johns Hopkins University School of Medicine Institutional Review Board, and all participants provided written informed consent.

### 2.1. Treatment Setting

The treatment setting for the parent study was the Center for Addiction and Pregnancy (CAP) [14, 16], a comprehensive care setting located on the Johns Hopkins Bayview Medical Center campus in Baltimore, Maryland. 

### 2.2. Intervention Conditions

#### 2.2.1. Usual Care (UC) Condition

CAP provides addiction treatment (group and individual therapy and psychoeducation), methadone maintenance for opioid-dependence, medically assisted withdrawal for opioid-dependent patients declining methadone or patients not meeting current opioid dependence criteria, general medical management, obstetrical care, case management, psychiatric evaluation and treatment, and on-site child care. Maternal treatment begins with a seven-night stay on an assisted living unit (ALU) followed by intensive outpatient treatment.

#### 2.2.2. Reinforcement-Based Treatment (RBT) Condition

The RBT condition was comprised of all the elements of the UC condition *plus* the RBT treatment elements. Thus, participants in the RBT condition received individualized treatment planning, behavior graphing, as well as exposure to weekly recreational, vocational, and peer reinforcement groups [17].

RBT participants were escorted by study counseling staff to a women's-only recovery house on the ALU day of discharge. Transportation back to CAP the following morning was provided to all RBT participants to provide additional support for their initial treatment engagement. In order to minimize risk for relapse during a high-risk period, RBT participants were expected to attend CAP seven days a week during the first month of outpatient treatment. RBT individual counseling sessions were scheduled 2-3 times a week and included review of behavior graphs and elements of vocational counseling. Recreational activities included participating in arts and crafts activities at the recovery house in a group format, as well as organized community activities such as attendance at poetry readings and movies. Social club was offered on Fridays, at which time participants were served lunch, reviewed progress on and set recreational goals for the upcoming week, were given the opportunity to interact with non-drug-using peers, and received certificates of achievement for cumulative days in treatment.

### 2.3. Participants

Participants were recruited from CAP between September 2003 and November 2007. Inclusion criteria were CAP admission, minimum 18 years of age, single fetus, self-reported heroin or cocaine use in the past 30 days, completion of a 7-night inpatient stay on an ALU, and willingness to live in recovery housing or other drug-free housing. Exclusion criteria included gestational age of 35 or more weeks based on last menstrual period and sonogram, and/or a severe concomitant medical or psychiatric condition that would interfere with study consent or participation. 

Of the 128 CAP patients who signed informed consent and were randomly assigned to their respective UC or RBT condition, 39 left treatment shortly after providing their consent to participate in the study but before they could be informed of their treatment condition assignment. The remaining 89 participants were informed of their treatment condition and thus comprised the sample subject to analysis: RBT (*n* = 47) or TAU (*n* = 42).

### 2.4. Measures

All measures were administered at study treatment entry and at a one-month follow-up point, in order to assess the short-term effects of treatment.

#### 2.4.1. Addiction Severity Index (ASI)

The ASI [18] is a semistructured interview assessing both lifetime and past-30 day events and behaviors in seven areas (Medical, Employment, Drug, Alcohol, Legal, Family/Social, and Psychiatric). It has excellent inter-rater and test-retest reliability, as well as concurrent and predictive validity. Standardized interviewer training and on-going inter-rater reliability are described elsewhere [12].

#### 2.4.2. Baltimore Risk Assessment Battery (BRAB)

A modified HIV Risk Assessment Battery (RAB) [19], the BRAB [20], was employed to measure HIV sexual and drug-use risk. The RAB evaluates HIV sexual and drug-risk behaviors over the past month and six months and has established reliability and validity [19–21]. The BRAB includes 9 items that measure HIV sexual-risk and 11 items that assess HIV drug-risk behavior. The sexual and drug-use risk scores can range between 1–25 and 0–29, respectively, with higher scores indicating greater HIV-risk behavior.

### 2.5. Risk Markers

A primary focus of this study was on risk markers that might moderate change over time. Nine potential risk markers were chosen a priori for examination. Information on (1) Age, (2) Race, and (3) Estimated Gestational Age was determined at study entry. Estimated gestational age was determined by number of weeks from last reported menstrual period. Moreover, six questions in the ASI was used to measure three potential lifetime and three potential current sexual and/or drug-risk markers: Lifetime—(4) Number of Times Treated for Substance use, (5) History of Prostitution Charges (yes versus no), and (6) History of Serious Depression (yes versus no); Past 30 days—(7) Recent Heroin Injection (yes versus no), (8) Currently Living in a Private Residence (yes versus no), and (9) Significant Other Who Currently Uses Drugs (yes versus no).

### 2.6. Statistical Analyses

A general linear mixed model (GLMM) analysis was conducted separately for each of the two BRAB scales, assuming a normal distribution, a compound symmetric error structure, and error degrees of freedom determined by the Kenward-Roger method (sometimes leading to fractional error *df*). The statistical model included the main effects for Treatment Condition (RBT versus TAU) and Time (baseline versus 1-month followup) and the 9 risk markers, as well as the interaction of Treatment Condition and each of the 9 risk markers with Time. These latter effects were included to determine the extent to which Treatment Condition and/or the 9 risk markers moderated change from baseline to 1-month followup. Tests of simple main effects were employed following a significant interaction. In the case of mean differences for categorical predictors, interpretation focused on model-derived least squares means and their standard errors, while in the case of continuous predictors, interpretation focused on the unstandardized partial regression coefficients and their standard errors.

## 3. Results

### 3.1. Participant Characteristics

Basic demographic information about the participants can be found in Table 1 along with information about their drug use history prior to study entry, as well as information from 6 items from the ASI. These 6 items, along with age, race, and estimated gestational age, all measured at study entry, served as the 9 risk markers used in the statistical model to examine the role these markers might play in moderating responsiveness to treatment.

Examination of the data from which this information was derived suggests that the participants spanned a large age range (from 18 to 42, inclusive), had likely not completed high school (only 20 women had an education beyond high school), and were entering treatment relatively late in their pregnancy (56% of the women were entering the study at 20 or more weeks of estimated gestational age). In addition, only 8 of the women were employed, and 11 married. Their recent drug use activity suggests a broad range of use of both heroin (with *n* = 31 having 0 days and *n* = 32 having 30 days of heroin use) and cocaine (with *n* = 24 having 0 and *n* = 22 having 30 days of cocaine use) and little use of other opioids or alcohol.

A review of the 6 risk markers from the ASI suggests a broad range of risk: 16 of the women had never before been treated for substance use, 67 of the women had never been charged with prostitution, 60 did not have a partner that used drugs, 62 were living in a private residence, and 62 had no history of intravenous drug use. However, 47 admitted to experiencing serious depression in their lifetime.

### 3.2. HIV Sexual and Drug-Use Risk

Neither the main effect for Treatment Condition nor Time nor their interaction was significant for either BRAB sexual or drug-risk scores (all *P*s > .27). Thus, presentation and discussion of the remaining results focus on the findings for 9 risk markers and their interactions with the Time effect. (There was only a single main effect for a risk marker: Recent Heroin Injection in the case of HIV drug-risk; however, because the Recent Heroin Injection × Time interaction effect was significant, interpretation focused on the interaction effect and not the Recent Heroin Injection effect.)

### 3.3. HIV Sexual Risk

For HIV sexual risk, there were 3 significant risk factor × Time interactions: currently Living in a Private Residence × Time (*F*(1,45.5) = 4.9, *P* < .04), History of Prostitution Charges × Time (*F*(1,4.07) = 6.2, *P* < .02), and Lifetime Number of Times Treated for Substance use × Time (*F*(1,35.6) = 5.2, *P* < .03).

**Figure 1:**
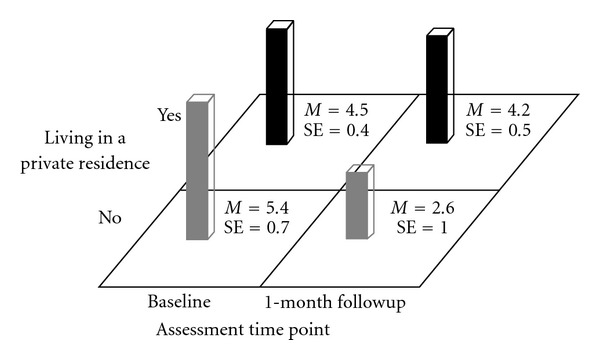
Block Chart of Currently Living in a Private Residence × Time Means for Baltimore Risk Assessment Battery Sex-Risk Scores.

Mean BRAB HIV sexual risk scores were not significantly different at baseline between participants who were and were not in stable housing (*P* > .2). However, participants who were in stable housing at baseline did not lower their mean BRAB HIV sexual risk scores from baseline to 1-month followup (*M* = 4.5 (SE = .4) versus *M* = 4.2 (SE = .5), *F*(1,37.2) = .1, *P* > .7) but unstably housed participants did (*M* = 5.4 (SE = .7) versus *M* = 2.6 (SE = 1.0), *F*(1,45.9) = 6.5, *P* < .014) (see Figure 1). 

**Figure 2:**
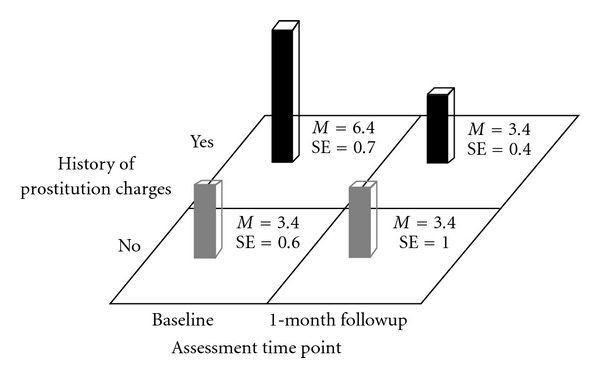
Block Chart of History of prostitution Charges × Time Means for Baltimore Risk Assessment Battery Sex-Risk Scores.

Participants with a history of charges for prostitution differed from participants without a history of charges for prostitution at baseline in terms of their mean BRAB HIV sexual risk scores (*M* = 6.4 (SE = .7) for the group with a history of charges for prostitution versus *M* = 3.4 (SE = .4) for the group with no history of prostitution, *F*(1,77.9) = 12.9, *P* < .001 but not at 1-month followup *M* = 3.4 (SE = .6) for the group with a history of prostitution versus *M* = 3.4 (SE = 1.0) for the group with no history of prostitution, *F*(1,81) = 0.0, *P* > .9. As would be expected, the test of the simple effects associated with Time within History of Prostitution Charges indicated that the group with a history of prostitution reduced their mean BRAB HIV sexual risk scores from baseline (*F*(1,41.1) = 7.6, *P* < .009) while the group with no history of prostitution did not reduce their mean BRAB HIV sexual risk scores (*F*(1,42.9) = 0.0, *P* > .9) (see Figure 2).

**Figure 3:**
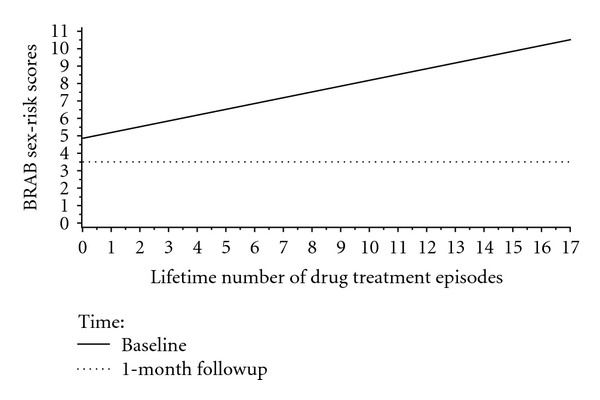
Plot of the Lifetime Number of Drug Treatment Episodes × Time Interaction for Baltimore Risk Assessment Battery Sex-Risk Scores.

Finally, tests of the simple main effects of Lifetime Number of Times Treated for Substance use within Time revealed that the number of times treated for substance use was directly related to BRAB HIV sexual risk at baseline (*b* = .3 (SE = .1), *t*(77.7) = 3.1, *P* < .004) but not at 1-month followup (*b* = .0 (SE = .1), *t*(80.3) = .1, *P* > .9) (see Figure 3).

### 3.4. HIV Drug-Use Risk

For HIV drug-use risk, there were 5 significant risk marker × Time interactions, involving Age (*F*(1,32.1) = 5.1, *P* < .04), Estimated Gestational Age at Study Entry (*F*(1,31.3) = 7.0, *P* < .02), Lifetime Number of Times Treated for Substance Use (*F*(1,28.9) = 21.8, *P* < .001), Lifetime History of Serious Depression (*F*(1,31.6) = 9.6, *P* < .005), and Recent Heroin Injection History (*F*(1,31.2) = 6.8, *P* < .02).

**Figure 4:**
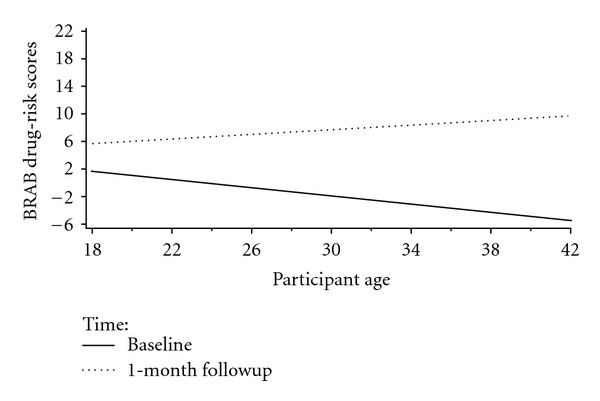
Plot of Participant Age × Time Interaction for Baltimore Risk Assessment Battery Drug-Risk Scores.

Age was nonsignificantly negatively related to BRAB HIV drug-use risk at baseline (*b* = −.1 (SE = .1), *t*(65) = −1.2, *P* > .2) and nonsignificantly positively related to BRAB HIV drug-use risk at 1-month (*b* = .1 (SE = .1), *t*(81) = 1.1, *P* > .2), indicating that the relationship between age and HIV drug-use risk significantly changed its direction, with increasing age at baseline associated with decreased HIV drug-use risk and increasing age at 1-month followup associated with increased HIV drug-use risk (see Figure 4).

**Figure 5:**
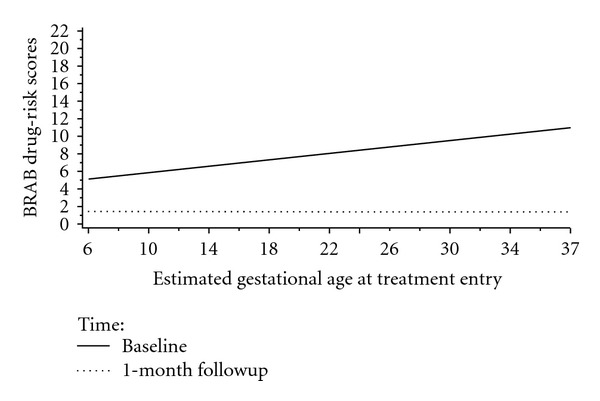
Plot of Estimated Gestational Age at Treatment Entry × Time Interaction for Baltimore Risk Assessment Battery Drug-Risk Scores.

Estimated gestational age was related to BRAB HIV drug-use risk at baseline (*b* = .2 (SE = .1), *t*(66) = 2.6, *P* < .02) but not at 1-month followup (*b* = −.0 (SE = .1), *t*(80.1) = −.1, *P* > .9) (see Figure 5). 

**Figure 6:**
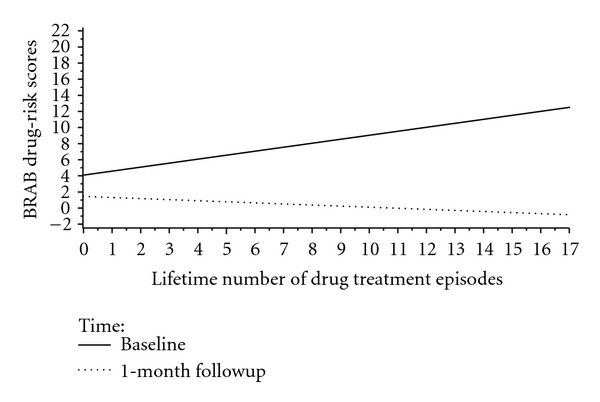
Plot of the Lifetime Number of Drug Treatment Episodes × Time Interaction for Baltimore Risk Assessment Battery Drug-Risk Scores.

Similarly, lifetime number of drug treatment episodes was related to BRAB HIV drug-use risk at baseline (*b* = .5 (SE = .2), *t*(64.1) = 2.9, *P* < .01) but not at 1-month followup (*b* = −.2 (SE = .2), *t*(73.1) = −1.3, *P* > .2) (see Figure 6). 

**Figure 7:**
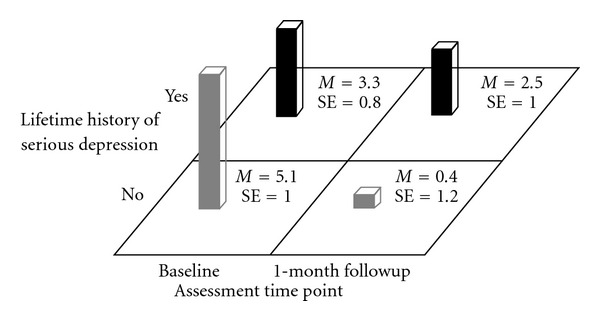
Block Chart of Lifetime History of Serious Depression × Time Means for Baltimore Risk Assessment Battery Drug-Risk Scores.

Participants without a history of depression had a significant decrease in their mean BRAB HIV drug-risk scores from baseline to 1-month followup (*M* = 5.1 (SE = 1.0) versus *M* = .4 (SE = 1.2), *F*(1,32.1) = 17.9, *P* < .001) while participants with a lifetime history of depression did not show such a decline in their mean BRAB HIV drug-risk scores from baseline to 1-month followup (*M* = 3.3 (SE = .8) versus *M* = 2.5 (SE = 1.0), *F*(1,30.7) = 1.1, *P* > .3) (see Figure 7).

**Figure 8:**
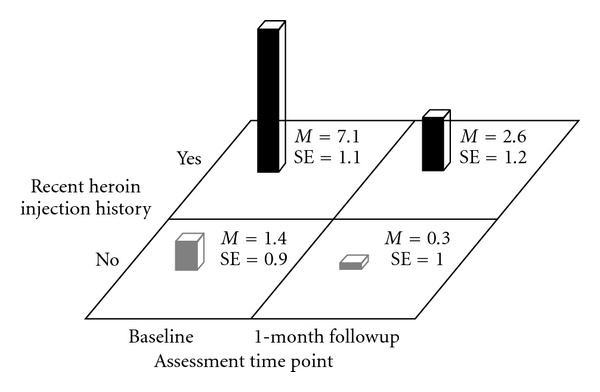
Block Chart of Recent Heroin Injection History × Time Means for Baltimore Risk Assessment Battery Drug-Risk Scores.

Finally, participants with a recent history of heroin injection were able to dramatically reduce their mean BRAB HIV drug-use risk from baseline to 1-month followup (*M* = 7.1 (SE = 1.1) versus *M* = 2.6 (SE = 1.2), *F*(1,30.9) = 17.5, *P* < .001) while participants without a recent history of heroin injection did not show such a decline in their mean BRAB HIV drug-risk scores from baseline to 1-month followup (*M* = 1.4 (SE = .9) versus *M* = .3 (SE = 1.0), *F*(1,32.2) = 1.4, *P* > .2). This pronounced difference in the reduction of mean BRAB HIV drug-use risk was primarily due to the fact that the two groups entered the study with quite different baseline mean BRAB HIV drug-use risk, *F*(1,65.2) = 19.8, *P* < .001 (see Figure 8).

## 4. Discussion

There are several strengths found in the present study. First, it is important to note that the sample of women entering the study was quite diverse not only in terms of their background characteristics such as age and race, but also in terms of their recent substance use history. Thus, findings should generalize to the larger population of substance-using pregnant women in this region.

Second, the fact that the Time main effect and Treatment Condition × Time interaction were nonsignificant for both HIV sexual and drug-use risk should not be viewed as a weakness in the study. It is certainly the case that both RBT and UC participants, on average, show little substantive short-term change from baseline, a disappointing finding. However, these results need to be seen in the context of the fact there were a number of risk markers that interacted with Time in predicting both HIV sexual and drug-use risk. These results strongly suggest the fact that there are multiple patient characteristics that moderate response to comprehensive treatment for substance use in pregnant patients. 

Indeed, study results demonstrate that similar to nonpregnant drug-dependent patients, some subgroups of pregnant patients receiving comprehensive drug treatment are able to show reductions in HIV sexual and drug-use risk behaviors. Comprehensive treatment may be especially beneficial for helping substance-using pregnant patients with histories of prostitution charges and/or current unstable housing to reduce their sexual risk behavior. Similarly, comprehensive treatment may also be especially beneficial for helping nondepressed and current heroin injectors to reduce their HIV drug-use risk behaviors. 

It is also the case that the continuous risk markers of lifetime number of times treated for substance use (for both HIV sexual and drug-use risk) and estimated gestational age at study entry (for HIV drug-use risk) significantly predicted HIV risk at baseline but not at 1-month followup. These findings should not be interpreted as supporting the conclusion that these risk markers failed to predict response to treatment—if that were the case, the interactions involving these risk markers would be nonsignificant. Instead the failure to predict likely reflects the fact that these risk markers are predictive of sexual and/or drug-use risk without exposure to comprehensive drug-abuse treatment, but that these risk markers are no longer operative in predicting risk following treatment. These results are encouraging, and merit further examination and replication.

Several limitations deserve mention. The first limitation is the fact that the present findings are based on data that were collected as a part of a larger behavioral trial where the number of potential participants excluded was relatively large, thus reducing the generalizability of the results. Furthermore, the parent study did not have as its primary goal the assessment of change in HIV sexual and drug-use risk. Therefore, the HIV sexual risk and drug-use risk behaviors were measured with a general instrument, and one not targeted specifically to substance-using pregnant women. The instrument also lacked specificity to detailed drug and sexual risk behavior. Future studies of substance-using pregnant women should include more sensitive and fine-grained measures of sexual and drug-use risk behaviors to more fully inform the knowledge base. Likewise, future HIV risk research with this population should include a measure examining specific gender and power risks in order to more accurately determine HIV sexual and drug-risk behaviors. Moreover, it may have been that the length of treatment was insufficient to produce changes that would have been detected by the risk markers under examination. Finally, the risk markers were not chosen as an integral part of the original study design; rather, they were chosen based on previous research in nonpregnant substance-using populations. It may well be the case that risk markers other than the ones included in the present study may prove more important than those markers examined in the present research. 

In summary, results of this secondary analysis study demonstrate several notable findings that inform both the research on and clinical treatment of pregnant women for substance use disorders. Finally, these results affirm the importance of comprehensive treatment for not only improving maternal and neonatal outcomes, but extending the importance of comprehensive care for reducing sexual and drug-use risk behaviors.

##  Conflict of Interests

The authors declare no conflict of interests.

## Figures and Tables

**Table 1:** Baseline participant characteristics and risk-factor status (*N* = 89).

Characteristic	*M*	SD
Age in years	30.7	5.9
Years of education completed	11.6	1.5
Estimated gestational age at study entry	20.5	7.9

	**%**	

Race		
Black	57.3%	
White	42.7%	
Unemployed	90.9%	
Not currently married	87.5%	
On probation or parole	33.0%	

Self-reported substances used in the 30 days prior to treatment entry		

	*M*	SD

Cocaine	13.4	12.2
Heroin	14.9	13.6
Other opioids	0.8	3.2
Alcohol	2.3	5.6

Risk Markers	*M*	SD

Lifetime number of treatments for drug abuse	3.1	3.1

	**%**	

Lifetime history of serious depression [yes]	53.4	
Lifetime history of prostitution charges [yes]	24.7	
Currently living in a private residence [yes]	70.5	
Recent heroin injection history [yes]	30.3	
Significant other currently uses drugs [yes]	23.1	

Note. Six questions on the ASI were used to measure the 6 risk markers listed at the bottom of the table. In addition to these 6 markers, the 3 baseline characteristics of age, race, and estimated gestational age at study entry were examined as possible risk markers.
